# Health and Wellness Characteristics of Employees Enrolled in a Workplace Wellness Study in the United Arab Emirates: A Descriptive Analysis of a Pilot Study

**DOI:** 10.7759/cureus.62294

**Published:** 2024-06-13

**Authors:** Alshafi Mohammad, Marília Silva Paulo, Salama Al Hosani, Omar Al Jabri, Zain Al Yafei, Sonali Datta, Erik Koornneef

**Affiliations:** 1 Clinical Trials Unit, Sheikh Shakhbout Medical City, Abu Dhabi, ARE; 2 Research and Innovation, Pure Health, Dubai, ARE; 3 Public Health, College of Medicine and Health Sciences, United Arab Emirates University, Al Ain, ARE; 4 Comprehensive Health Research Center, NOVA Medical School, Universidade NOVA de Lisboa, Lisbon, PRT; 5 Health Operations Management, Ambulatory Health Services, Abu Dhabi, ARE; 6 Pathology and Laboratory Medicine, PureLab, Abu Dhabi, ARE; 7 Research and Innovation, Pure Health, Abu Dhabi, ARE

**Keywords:** workplace wellness, well-being, health, employees, united arab emirates

## Abstract

Introduction: Modifiable health behaviors have the power to increase (or decrease) the risk of chronic diseases, impacting a population’s health. Health and wellness programs can potentially play a major role in initiating and supporting positive changes in health behaviors, which may lead to reducing the risk of premature mortality. A better understanding of the health and well-being status of the population is crucial to the design of proper and effective interventions. This pilot study aimed to describe the health and well-being status of a cohort of employees in the United Arab Emirates (UAE).

Methods: This pilot study reports the demographic characteristics, body composition, cardiovascular fitness, functional fitness, biological age, and well-being of employees from a large health sector company enrolled in a workplace wellness study in the UAE. Employees were invited to participate in an intervention that was designed to validate the efficacy of weekly health and wellness challenges. Descriptive statistics were used to describe the employees' distribution.

Results: Of the 123 selected, 116 employees participated in the study. The mean age of participants was 39.2 years old, 80% of them were non-Emirati, and the majority were from Middle-Eastern and South Asian ethnicities. The prevalence of overweight, obesity, hypercholesterolemia, hyperlipidemia, prediabetes, and diabetes was 35%, 29%, 34%, 79%, 30%, and 7%, respectively. Almost half of the participants (47%) were prehypertensive for systolic blood pressure (BP), 80% had the fitness category of poor-very poor, and the majority (60%) reported exercising <150 minutes/week. The mean functional fitness score was 12.2 points, which indicated an increased risk of injury with physical activity.

Conclusion: The findings of this pilot study suggest that despite the advancements in healthcare in the UAE, several key preventable risk factors are still prevalent in its population. The introduction of comprehensive health and wellness programs at a broader scale holds the potential to facilitate the adoption of healthier lifestyle behaviors, thereby contributing to improvements in the overall quality of life across the population.

## Introduction

Chronic diseases have been considered a global epidemic for more than one decade, resulting in the death of 41 million people each year, equivalent to 74% of all deaths globally [1]. Non-communicable diseases or chronic diseases are a well-defined hazard for one's health and result from a combination of genetic, physiological, environmental, and behavioral factors [1,2]. These diseases can be preventable or delayed by empowering the population to modify their behaviors like tobacco use, physical inactivity, unhealthy diet, and alcohol abuse, ultimately leading to the adoption of a healthy lifestyle.

There is no consensus among the scientific community on the proportion of contributing factors for the development of disease; study results vary slightly according to their sampling population. However, it is estimated that behaviors account for 36% to 50% of contributing factors for health outcomes [2].

The United Arab Emirates (UAE) has a unique population of 9.9 million people (2021), where 8.8 million (89%) are expats from more than 200 nationalities [3]. This population uniqueness is also reflected in the age pyramid of the country with a high proportion of people aged 25-54 (66%), followed by children aged 0-14 years old (15%) [3]. The UAE has a high rate of chronic diseases, which are projected to increase as the population ages [4]. Chronic diseases are also the cause of most deaths; ischemic heart disease, stroke, chronic kidney disease, diabetes, chronic obstructive pulmonary disease, and cancer are among the top 10 causes of death and disability combined [5]. Obesity, hypertension, diabetes, and smoking have a high prevalence in the UAE and are under constant monitoring [6,7].

The UAE has a strong foundation in place to address the challenge of chronic diseases [4,8]. To contribute to the UAE’s aim of improving the health outcomes of the population, it is also important to have longitudinal studies that reflect the unique characteristics of the population. There is no evidence of a similar population health study in the UAE, comprising this variety of outcomes and social determinants of health. This is important to determine the baseline state of health of the population we are aiming for.

Accordingly, this study aimed to describe the health and well-being status of employees of a large healthcare company enrolled in a workplace wellness program in the UAE. We characterized participants by evaluating their demographics, body mass composition, cardiovascular fitness, functional fitness, blood biomarkers, and self-reported overall health, and well-being status. This study serves as a pilot for a larger, future investigation exploring the effect of a health and wellness program intervention on a more extensive group of employees. 

This article was previously posted to the Research Square preprint server on August 03, 2023.

## Materials and methods

Ethics approval and consent to participate

This study was approved by the Institutional Review Board (IRB) of Abu Dhabi Health Services Co. (SEHA) on 25th October 2022 under the application number SEHA-IRB-45 and performed in accordance with relevant guidelines and regulations including the Declaration of Helsinki. Written informed consent was obtained from all the participants. This is an observational descriptive study reported following the STROBE Statement [9].

Study setting

This study was conducted by a specialized team from the Research and Innovation department at Pure Health (Dubai, UAE), one of the largest companies in the UAE health sector.

Participants

Eligibility Criteria

To be eligible to participate in the three-month (December 2022 - March 2023) workplace wellness study, participants had to meet the following criteria: (a) employed at the company; (b) aged 18 years and older; (c) available during the study duration; (d) willing to commit to all the procedures and activities relevant to the study during its three months period; and (e) confirm participation through the informed consent form. The exclusion criteria included (a) unsure or unable to fully commit to all the procedures and activities relevant to the study; (b) advised not to exercise by a licensed healthcare practitioner, regardless of the exercise type or intensity; (c) having a medical condition(s) that would prevent exercising, regardless of the exercise type or intensity; (d) pregnant or breastfeeding; (e) severe illnesses; (f) severe injury in the joints or the back; (g) planning major surgical procedures or other major treatment during the study period; (h) participating in any other health promotion program.

After obtaining agreement, participants were asked to complete a self-reported overall health and well-being survey, give blood samples for blood biomarker assessment, and undergo body mass composition assessment, functional fitness assessment, and cardiovascular fitness assessment.

Variables, data sources, and measurements

The variables collected for the purpose of this study are clustered by outcomes. Table 1 describes, for each variable of interest, the sources of data and the details of the assessment method/measurements used.

**Table 1:** Outcome variables and measurements/data sources BMI: body mass index; HDL: high-density lipoprotein; LDL: low-density lipoprotein, DHEAS: dehydroepiandrosterone sulfate; GGT: gamma-glutamyl transferase; HbA1c: hemoglobin A1c; hs-CRP: high-sensitivity C-reactive protein; SHBG: sex hormone binding globulin; TIBC: total iron binding capacity; RDW-CV: red cell distribution width - coefficient of variation; MCH: mean corpuscular hemoglobin; BP, blood pressure; HR, heart rate

Outcomes	Variables	Measurements/data sources
Socio-demographic characteristics	Emirate, age, sex, nationality, ethnicity, marital status, number of children, education level, smoking status, and physical activity	Self-reported survey supervised
Body composition	Height, weight, BMI, waist circumference, body fat percentage	Physical assessment
Functional fitness	Deep squats, hurdle steps, in-line lunges, ankle clearing tests, shoulder mobility, shoulder clearing tests, active straight leg raises, trunk stability push-ups, extension clearing tests, rotatory stability, and flexion clearing tests	Physical assessment
Cardiovascular fitness	Systolic BP, diastolic BP, resting HR, one-mile Rockport walking test, walk completion HR, predicted VO2 max, fitness percentile/category	Physical assessment
Biomarkers for health status	Total cholesterol, HDL, LDL, total cholesterol/HDL ratio, triglyceride, glucose fasting	Phlebotomy
Biomarkers for biological age	Albumin, calcium, DHEAS (females), fasting glucose, GGT, HbA1c, hs-CRP, LDL, SHBG (males), testosterone (males), TIBC, triglycerides, red blood cells, RDW-CV, hematocrit, monocytes, neutrophils, MCH, lymphocytes, eosinophils, basophils	Phlebotomy
Well-being	Physical activity, sleep, fruit and vegetable consumption, sweets and sugary beverages consumption, water drinking, stress levels, tobacco consumption, alcohol consumption, physical examination	Self-reported survey

Registered nurses and licensed nutritionists monitored the body composition assessment. Functional fitness was assessed under the supervision of a certified fitness instructor and according to Cooper Health Institute methods, which is a simple method to identify movement patterns asymmetries or deficiencies needed for everyday physical activity. Cardiovascular fitness assessment was performed under the supervision of a licensed cardiologist. The blood samples were collected by registered nurses to evaluate blood biomarkers to obtain a description of both the overall health status and the biological age [10]. All personnel involved in taking these measurements were fully trained, licensed, and registered, following standardized protocols to ensure consistency and accuracy.

Study size

The sample size for this pilot study was determined based on the sample sizes typically observed in pilot studies reported in the literature. Given the descriptive nature of this pilot research, a voluntary, convenience sample approach was selected, with employees invited to participate in the study through online invitations. The online invitations included a summary covering the nature of the study, the objectives of the study, the study procedures and activities, and the eligibility criteria.

Quantitative variables

Body Composition

Height and weight were measured in meters (m) and kilograms (kg), respectively, using the medical weighing scale with a stadiometer. Waist circumference was measured in centimeters (cm) using measuring tape. The body fat percentage was measured using a handheld body fat analyzer, and body mass index (BMI) was calculated using weight in kg divided by the square of height in meters (kg/m^2^). BMI was classified into four categories: underweight (<18.5, healthy weight (18.5-24.9), overweight (25.0-29.9), and obesity (≥30.0).

Functional Fitness

Functional fitness was evaluated using a functional movement screen (FMS) test kit, which was designed to provide a simple grading system to assess participant movement. The functional fitness test included movements, such as deep squats, hurdle steps, in-line lunges, shoulder mobility, active straight leg raises, trunk stability push-ups, and rotatory stability. The test scoring ranged from 0 to three points, with four possible scores that a person could get. A score of 0 indicated pain during the movement. A score of one indicated an inability to complete the full movement properly or an inability to get into the correct position to execute the movement. A score of two indicated completing the movement with compensation. A score of three indicated the movement was optimal without compensations, with the sum creating a total score ranging from 0 to 21 points (a total score of 14 or lower indicated 1.5 to 2.0 times higher risk for injury than those with a score higher than 14) [11]. Other variables from the same outcome were dichotomous variables assuming the values of positive or negative, such as the shoulder clearing test, extension clearing test, and flexion clearing test. The ankle clearing test was also a qualitative variable assuming the category beyond, within, or behind, while the area requiring improvement assumed the category of mobility or stability.

Cardiovascular Fitness

Cardiovascular fitness outcome was composed of numerical variables and a categorical variable. The numerical variables included systolic and diastolic blood pressure (BP), resting and exercise heart rate (HR), one-mile Rockport walking test, predicted VO2 max, and fitness percentile. Systolic and diastolic BP was measured using a mobile BP/vital signs monitor (normal values range: <120/80 mm Hg, prehypertension: 120-139/80-89 mm Hg, and hypertension: ≥140/90 mm Hg). Resting HR and exercise HR (at the completion of the one-mile Rockport test) were measured (beats per minute, BPM) using an HR monitor. One-mile Rockport walking test was conducted using a commercial treadmill, and walk completion time was measured in both minutes and seconds using a stopwatch. Participants were asked to warm-up for around five to 10 minutes before the walk. The predicted VO2 max (mL/kg/min) was measured to predict an individual’s aerobic capacity using information, such as age (years), gender (male/female), body weight (kg), walking completion time (minute and seconds - expressed as a percentage) and exercise HR (BPM). The formula for calculating VO2 max using the one-mile walk test was 32.853-(0.0769×weight)-(0.3877×age)+(6.315×gender (male=1, female=0)-(3.2649×time)-(0.1565×HR) [12]. The fitness percentile (%) was generated from the predicted VO2 max score, which represented the overall fitness.

Blood Biomarkers for Health Status and Biological Age

Blood samples were collected through venipuncture. Per participant, 26 mL of blood was collected in vacutainer tubes to analyze biomarkers for health status and biomarkers relevant to biological aging [10]. The biomarkers for health status assessment included total cholesterol (mmol/L), high-density lipoprotein (HDL) (mmol/L), low-density lipoprotein (LDL) (mmol/L), total cholesterol/HDL ratio, triglyceride (mmol/L), and glucose fasting (mmol/L). The biomarkers for biological age assessment included albumin (g/L), calcium (mmol/L), dehydroepiandrosterone sulfate (DHEAS) (mmol/L; females), fasting glucose (mmol/L), gamma-glutamyl transpeptidase (GGT) (IU/L), hemoglobin A1c (HbA1c) (%), high-sensitivity C-reactive protein (hs-CRP) (mg/L), LDL (mmol/L), sex hormone binding globulin (SHBG) (nmol/L; males), testosterone (nmol/L; males), total iron binding capacity (TIBC) (micromol/L), triglycerides (mmol/L), red blood cells (x10^12^/L), red cell distribution width - coefficient of variation (RDW-CV) (%), hematocrit (L/L), monocytes (%), neutrophils (%), mean corpuscular hemoglobin (MCH) (%), lymphocytes (%), eosinophils (%), basophils (%). The biological age was calculated using previously described methods [10].

Well-Being

The well-being was assessed using a self-reported questionnaire (developed by the Cooper Aerobics Center (founded by Dr. Kenneth H. Cooper in 1971)), which identified possible areas of improvement to achieve total well-being. The 10-question assessment was composed of categorical variables assuming between three to five nominal categories and evaluated current habits and lifestyle behaviors, such as physical activity, sleep, fruit and vegetable consumption, sweets and sugary beverages consumption, water drinking, stress levels, tobacco consumption, alcohol consumption, and physical examination [13].

Statistical methods

A descriptive analysis was performed to report the demographic, social, and professional characteristics of the enrolled participants. Categorical data was described using percentages, and continuous data was described using mean and standard deviation (SD) unless otherwise stated.

## Results

Participants

An online invitation was sent to 1859 employees and 279 (15%) volunteered. From those, 123 were selected considering the proportion of sex and ethnicities of the country. The participants comprised medical staff, allied health professionals, administrative staff, technical staff, human resources staff, finance and accounting staff, marketing and public relations staff, legal and compliance staff, management, and executive staff. Additionally, participants represented various ethnic backgrounds, including Asian, Black/African/Caribbean, Latin American/Hispanic, Middle-Eastern (Arab), South Asian (Pakistani-Indian), and White-Caucasian (Table 2).

**Table 2:** Baseline socio-demographic characteristics ^*^Four missing participants

Socio-demographic variables		Number of participants (%)
Age (mean), years	39.2	
Emirate	Abu Dhabi	54 (47%)
	Dubai	62 (53%)
Sex	Male	59 (51%)
	Female	57 (49%)
Nationality	Emirati	23 (20%)
	Non-Emirati	93 (80%)
Ethnicity	Asian	25 (22%)
	Black/African/Caribbean	6 (5%)
	Latin/American/Hispanic	1 (1%)
	Middle-Eastern (Arab)	34 (29%)
	South Asian (Pakistani-Indian)	33 (28%)
	White-Caucasian	17 (15%)
Marital status	Single	34 (29%)
	Married	73 (63%)
	Divorced	8 (7%)
	Widowed	1 (1%)
Children*	0	38 (33.93%)
	1	22 (19.64%)
	2	31 (27.68%)
	3	13 (11.61%)
	More than 3	8 (7.14%)
Educational status*	High school certificate	2 (2%)
	Bachelor’s degree	51 (46%)
	Master’s degree	48 (43%)
	PhD	5 (4%)
	Other	6 (5%)
Chronic diseases*	Yes	13 (12%)
	No	99 (88%)

Descriptive data

The ratio of participants living in Abu Dhabi and Dubai was evenly distributed, as well as the sex distribution with 51% males and 49% females. The mean age of participants was 39.2 years old, and the largest majority was non-Emirati (80%). Most of the participants were from Middle-Eastern and South Asian ethnicity and married. The majority of the participants (88%) reported not to have any chronic diseases (Table 2).

Main results

**Figure 1:**
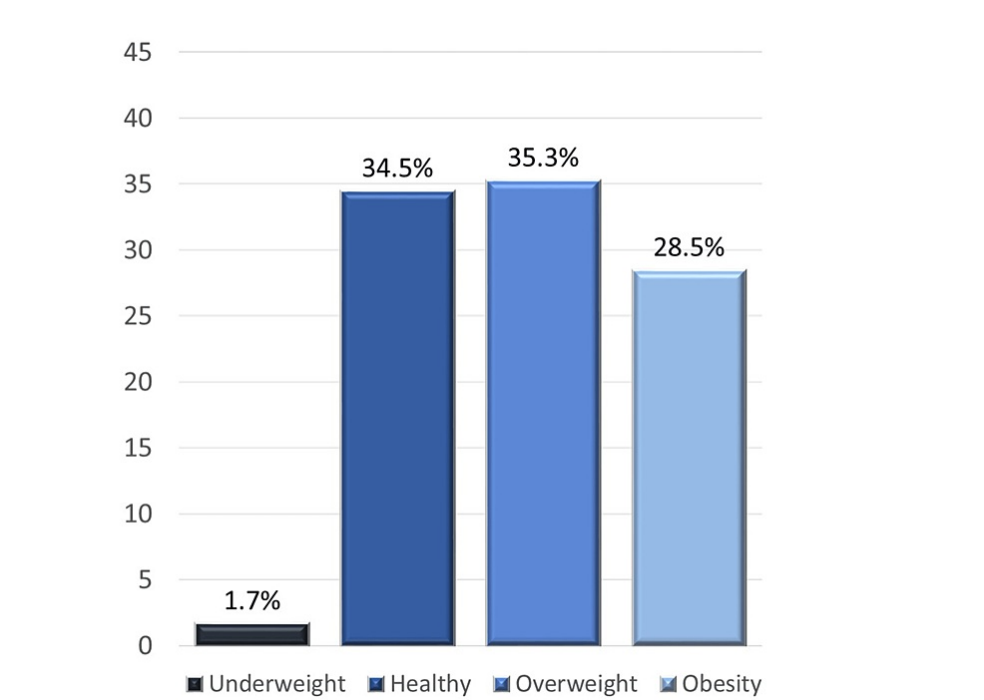
BMI distribution of participants BMI, body mass index

The mean BMI of the participants was 27.3 kg/m^2^. The majority of both men and women were overweight (28.0 vs. 26.6 kg/m^2^) but the BMI ranged from underweight (1.7% of participants had BMI <18.5 kg/m^2^) to obesity (28.5% had BMI >30.0 kg/m^2^; Figure 1). 

The waist circumference was 90.1 cm in men and 86.0 cm in women. According to the British Heart Foundation, the waist circumference of men was considered in the low-risk category compared to women’s waist circumference, which was considered in the high-risk category. The body fat percentage in men and women was 24.3 and 32.3, respectively, which was mildly above average range for women according to the American Council on Exercise (Table 3) [14].

**Table 3:** Body composition variables of participants, stratified by gender All variables were analyzed for 116 participants

Body composition variables		Mean±SD	Range
Height (cm)		169.4±8.8	148-188.4
	Men	175.5	148-188.4
	Women	163.3	148-175
Weight (kg)		78.5±17.2	43.9-123.4
	Men	86.4	64.5-123.4
	Women	70.8	43.9-109.8
BMI (kg/m^2^)		27.3±5.2	18.1-41.9
	Men	28.0	19.6-38.0
	Women	26.6	18.1-41.9
Waist circumference (cm)		92.1±15.1	63-150
	Men	90.1	78-132
	Women	86.0	63-150
Body fat percentage		28.2±8.7	8.2-47.2
	Men	24.3	8.2-39.1
	Women	32.3	13.6-47.2

Most of the participants have completed the functional fitness exercises with compensation (median scores of two). The mean score from all participants was 12.2 points, which indicated an increased risk of injury with physical activity (Table 4).

**Table 4:** Functional fitness assessment scores of participants All variables were analyzed for 116 participants.

Functional fitness scores		Mean±SD	Median score	Range	Number of participants (%) with a median score
Deep squat final score		1.9±0.7	2	0-3	81 (70%)
Hurdle step final score		1.6±0.6	2	0-3	68 (59%)
In line lunge final score		1.7±2.4	2	0-3	73 (63%)
Ankle clearing test	Left	-	Within	Behind, beyond, within	61 (53%)
	Right	-	Within	Behind, beyond, within	61 (53%)
Shoulder mobility		2.0±0.9	2	0-3	37 (32%)
Shoulder clearing test	Negative	-	-	-	112 (97%)
	Positive	-	-	-	4 (3%)
Active straight leg raise		2.1±0.7	2	0-3	66 (57%)
Trunk stability push-up		1.6±0.7	2	0-3	58 (50%)
Extension clearing test	Negative	-	-	-	109 (94%)
	Positive	-	-	-	7 (6%)
Rotatory stability		1.3±0.5	1	0-3	81 (70%)
Flexion clearing test	Negative	-	-	-	110 (95%)
	Positive	-	-	-	6 (5%)
Area requiring improvement	Mobility	-	-	-	27 (23%)
	Stability	-	-	-	89 (77%)
Total Score (0 to 21)		12.2±2.4	12	7-21	22 (10%)

The mean systolic and diastolic BP was 124.1 and 77.6 mm Hg, respectively. Almost half of the participants were prehypertensive (47%). The mean for a walk completion HR was 134 BPM, which was considered normal for adults (between 80 and 170 BPM). The predicted VO2 max of participants was 25.5 mL/kg/min, which was poor according to the American College of Sports Medicine. Moreover, three-quarters of the participants were in the fitness category of poor (Table 5).

**Table 5:** Cardiovascular fitness variables of participants ^*^Four missing participants. BP, blood pressure; HR, heart rate

Cardiovascular fitness variables		Mean (±SD)	Number of participants (%)
Systolic BP, mm Hg	Mean	124.1±13.5	
	Normal	-	46 (40%)
	Prehypertension	-	55 (47%)
	Hypertension	-	15 (13%)
Diastolic BP, mm Hg	Mean	77.6±9.8	
	Normal	-	67 (57.8%)
	Prehypertension	-	39 (33.6%)
	Hypertension	-	10 (8.6%)
Resting HR (BPM)		86.3±15.2	-
Walk completion HR (BPM)		134.0±20.2	-
Predicted VO2 max (mL/kg/min)		25.5±14.7	-
Fitness category/percentile (%)^*^	Excellent: 76 to 100%	-	6 (5%)
	Good: 56 to 75%	-	9 (8%)
	Fair: 36 to 55%	-	8 (7%)
	Poor: 16 to 35%	-	13 (12%)
	Very poor: 0 to 15%	-	76 (68%)

The mean total cholesterol and LDL values were 5.1 and 3.2 mmol/L, respectively. Blood test results showed that 34% of the participants had high total cholesterol and 79% had high LDL levels. The mean HbA1c of participants was 5.9%. Prediabetes was found in 30% and diabetes in 7% of the participants (Table 6). The mean biological age was assessed to be 45.1 years, which was six years older than the mean chronological age.

**Table 6:** Blood biomarkers of participants Prediabetes: HbA1c ≥ 5.7% and <6.5%; diabetes: HbA1c ≥ 6.5%. Analysis for 116 participants. HDL: high density lipoprotein; LDL: low-density lipoprotein; HbA1c, hemoglobin A1c

Biomarkers for health variables	Mean ± SD	Number of participants (%)
Total cholesterol, mmol/L	5.1±1.0	-
Elevated total cholesterol (>5.20 mmol/L)	-	40 (34%)
HDL, mmol/L	1.35±0.4	-
LDL, mmol/L	3.2±0.9	-
Elevated LDL (>2.41 mmol/L)	-	92 (79%)
Triglyceride, mmol/L	1.1±0.6	-
Glucose fasting, mmol/L	5.3±1.3	-
HbA1c (%)	5.9±0.8	-
Prediabetes	-	35 (30%)
Diabetes	-	8 (7%)

More than half of the participants reported exercising less than 150 minutes per week, which did not meet the global recommendations of at least 150 minutes of moderate-intensity activity per week. Half of the participants reported sleeping six or more hours per day, 65% had two portions of fruits or vegetables per day, and 11% limited the consumption of sugary food and drinks almost all the time. A few (17%) employees felt stressed or very stressed, 10% reported smoking, and only 13% consumed alcoholic beverages; among them, two had more than 10 drinks per week. Half of the participants underwent a complete physical examination with a physician within a one-year period (Table 7).

**Table 7:** Self-reported well-being description of participants *Missing data of 11 participants.

Well-being variables		Number of participants (%)	Comparison with UAE population
On average, how many minutes of physical activity do you get per week?	Less than 60 minutes	35 (30%)	Physical inactivity was 23.3% before the lockdown due to the COVID-19 pandemic and 32% during the lockdown [15]. More than 150 minutes of weekly activity in migrants before 5 years was 54% [16].
	60-90 minutes	16 (14%)	
	90-120 minutes	14 (12%)	
	120-150 minutes	6 (5%)	
	More than 150 minutes	45 (39%)	
On average, how many hours of sleep do you get per 24 hours?	Less than 4 hours	0 (0%)	A cross-sectional study of 494 participants reported that 81% had poor sleep [17].
	4-5 hours	19 (16%)	
	5-6 hours	34 (29%)	
	6-7 hours	40 (35%)	
	More than 7 hours	23 (20%)	
On average, how many servings of fruits and vegetables do you eat per day?	0-2 servings	75 (65%)	42% of medical students reported consumption of fruits and vegetables [18].
	2-4 servings	39 (33%)	
	5-9 servings	2 (2%)	
On average, how often do you limit sweets and sugary beverages?	Not at all	23 (20%)	A cross-sectional survey found that 92% of university students have at least one daily energy drink [19].
	Rarely	36 (31%)	
	Sometimes	44 (38%)	
	Most of the time	13 (11%)	
	Almost always	0 (0%)	
On average, how many cups of water do you drink per day?	1 cup or less	2 (2%)	No comparable data found.
	2-3 cups	9 (8%)	
	4-6 cups	31 (26%)	
	6-7 cups	23 (20%)	
	8 or more cups	51 (44%)	
How stressed do you feel on average?	Not stressed at all	8 (6.9%)	No comparable data found.
	A little stressed	45 (38.8%)	
	Somewhat stressed	44 (37.9%)	
	Stressed	17 (14.7%)	
	Very stressed	2 (1.7%)	
How well do you feel you manage your stress?	Not well at all	8 (6.9%)	No comparable data found.
	Somewhat well	46 (39.7%)	
	Well	37 (31.9%)	
	Very well	22 (18.9%)	
	Extremely well	3 (2.6%)	
Do you use any tobacco products?	Yes	12 (10%)	Tobacco smoking prevalence is 36% for men [20]. 21% of medical students reported ever smoked [18].
	Occasionally	16 (14%)	
	Not in the past 12 months	0 (0%)	
	Not in the past 24 months	0 (0%)	
	Not ever	88 (76%)	86% of medical students reported never consumed alcoholic drinks [18].
How many alcoholic beverages do you drink per week?^*^	0-2	92 (79%)	
	3-6	0 (0%)	
	7	11 (9%)	
	7-10	0 (0%)	
	More than 10	2 (2%)	
When was your last complete physical examination with a physician?	It has been more than 10 years	15 (13%)	No comparable data found.
	Within the past 5 years	12 (10%)	
	Within the past 3 years	29 (25%)	
	Within the past year	60 (52%)	

## Discussion

Key results

The population distribution of employees in this study was similar in terms of the country distribution of expatriates and their ethnicities [21,22]. The country's population is largely constituted of males who come to work in industries and construction sectors, very similar to other Gulf cooperation countries [4]. Moreover, the UAE is host to a larger number of expatriates, making up 88% of the entire population [3]. These three key characteristics highlighted here demonstrate the challenges when conducting a country versus employee population analysis and comparison [7,16,21]. The present study took those country characteristics into consideration and attempted to include as diverse pool of employees as possible.

In terms of chronic diseases in the UAE, this study found a low prevalence of chronic diseases compared with previous studies; this can be explained by the average age of the employees. Chronic diseases account for more than 65% of deaths in the country and the public health priority areas in the last 10 years have been cardiovascular diseases, road injuries, cancers, and respiratory disorders [21,22].

The age of the cohort in this study was comparable with the age reported in previous studies of the UAE population with a similar distribution of overweight and obesity categories observed, 35% and 29%, respectively [16]. In a study of UAE nationals, overweight accounted for 30% of the population and obesity for 27%; similarity, the prevalence of overweight studied in a cohort of migrants was 30% and 17% for obesity [16,23].

Systolic and diastolic hypertension, 13% and 9%, in this study were lower than previously reported prevalence in Emirati individuals, which was found to be 22% overall; whereas, male South Asians living in the UAE had a higher prevalence of hypertension, reported to be 30% [23,24].

The results from the cardiovascular fitness and the functional fitness indicated poor fitness conditions and an increased risk of injury with physical activity; moreover, the asymmetry and/or deficiency in performing the movements indicated a general lack of physical activity in our cohort. We found that the activity levels for 61% of participants were below the global recommended levels of at least 150 minutes of moderate to vigorous physical activity per week [25]. These findings are in accordance with previous studies of the UAE population where the shortage of physical activity was similarly observed. A study of female UAE migrants found that 54% had moderate to vigorous activity during the week, which was higher compared to our findings (39%); however, the proportion of physically active people declined to 49% after five years [16]. Moreover, a survey of 1540 people showed that the rate of physical inactivity was 23.3% before the lockdown due to the COVID-19 pandemic and 32% during the lockdown [15]. The 2022 Report Card that utilized data from 2017 to 2021 to apprise the core physical activity indicators for children and adolescents of the UAE showed that just one in five (19%) individuals achieved the recommended amount of moderate to vigorous activity [26]. Although physical activity levels remain relatively low in the UAE for children and adolescents, their findings showed an improvement of 3% from the previous Report Card released in 2018 [27].

We observed a high prevalence of elevated LDL (79%) in our study population, which was higher than previously found among 44% of UAE nationals [23]. Furthermore, we observed a prevalence of prediabetes and diabetes among 30% and 7% of our study population, respectively. This has previously been reported to be between 7.7% among childbearing age women to 21.6%, which was reported to be the second highest rate in the region, according to the latest findings of meta-analysis [28,29]. Whereas, the prevalence of undiagnosed diabetes in the UAE population was reported to be 14.6% [21,30].

The population in our study reported a low use of tobacco products, 14%, in comparison with previous reports of Emiratis where the prevalence of smoking was found to be 36% among men and 3% among women [20].

Limitations

This study was a pilot trial for subsequent, larger-scale studies and we used a sample size aligned with the literature recommendation for pilot research. Moreover, employees were selected using a convenience sampling method. As a result, this may limit the generalizability of our findings and our cohort may not be fully representative of employees across the entire organization or the UAE healthcare sector. Moreover, this study may have healthy bias effects as employees who have answered the online invitation may have been more prone to be intrinsically motivated to participate in the study resulting in better health statistics for certain variables than previously reported in the general UAE population.

## Conclusions

The healthcare system in the UAE has witnessed remarkable advancements in the recent years. However, despite these advancements certain preventable risk factors continue to affect the population. This underscores the need for targeted health and wellness programs, which can have a significant impact on addressing these risk factors. This in turn can lead to healthier lifestyles and improve the quality of life among the UAE population. To engage employees more effectively in wellness programs, companies may consider offering gym memberships, providing access to on-site fitness facilities, organizing marathons and other physical activity events, and incorporating incentives for participation in wellness challenges. Encouraging individuals to lead more active lives by reducing sedentary behaviors and promoting physical activity can help decrease rates of obesity, cardiovascular diseases, and diabetes. By placing emphasis on preventive measures and empowering individuals to make healthier choices, the UAE can alleviate pressure on its healthcare system and allocate resources more effectively.

## References

[1] (2023). World Health Organization. Noncommunicable diseases. http://www.who.int/news-room/fact-sheets/detail/noncommunicable-diseases.

[2] McGovern L, Miller G, Hughes-Cromwick P (2014). Health policy brief: the relative contribution of multiple determinants to health. Health Affairs Health Policy Brief.

[3] EDS. UNITED ARAB EMIRATES POPULATION REPORT 2022 (2023). EDS. United Arab Emirates population report 2022. http://edsfze.com/united-arab-emirates-population-report-2022/.

[4] Paulo MS, Loney T, Lapão LV (2017). The primary health care in the emirate of Abu Dhabi: are they aligned with the chronic care model elements?. BMC Health Serv Res.

[5] Vos T, Lim SS, Abbafati C, Abbas KM (2020). Global burden of 369 diseases and injuries in 204 countries and territories, 1990-2019: a systematic analysis for the Global Burden of Disease Study 2019. Lancet.

[6] Mamdouh H, Hussain HY, Ibrahim GM (2023). Prevalence and associated risk factors of overweight and obesity among adult population in Dubai: a population-based cross-sectional survey in Dubai, the United Arab Emirates. BMJ Open.

[7] Mamdouh H, Alnakhi WK, Hussain HY (2022). Prevalence and associated risk factors of hypertension and pre-hypertension among the adult population: findings from the Dubai Household Survey, 2019. BMC Cardiovasc Disord.

[8] Koornneef E, Robben P, Blair I (2017). Progress and outcomes of health systems reform in the United Arab Emirates: a systematic review. BMC Health Serv Res.

[9] No I, Background I (2008). STROBE statement--checklist of items that should be included in reports of observational studies (STROBE initiative). Int J Public Health.

[10] Westerman K, Reaver A, Roy C (2018). Longitudinal analysis of biomarker data from a personalized nutrition platform in healthy subjects. Sci Rep.

[11] O'Connor FG, Deuster PA, Davis J, Pappas CG, Knapik JJ (2011). Functional movement screening: predicting injuries in officer candidates. Med Sci Sports Exerc.

[12] (2023). The One-Mile Rockport Walking Test | HFE. http://www.hfe.co.uk/resources/health-and-fitness-assessments/the-one-mile-rockport-walking-test.

[13] (2023). Cooper Quest - Dallas - Cooper Aerobics. http://www.cooperaerobics.com/Cooper-Fitness-Center-Dallas/For-Members-Only/Cooper-Quest.aspx.

[14] (2023). ACE Fit | Percent Body Fat Calculator. http://www.acefitness.org/resources/everyone/tools-calculators/percent-body-fat-calculator/..

[15] Al Sabbah H, Taha Z, Qasrawi R (2022). The impact of COVID-19 on physical (in)activity behavior in 10 Arab countries. Int J Environ Res Public Health.

[16] Shah SM, Silva Paulo M, Loney T, Nauman J, Govender RD, Govender D (2023). Association between acculturation and obesity among female migrants in the United Arab Emirates: a population-based study. Ibnosina J M Bio Sci.

[17] Abedalqader F, Alhuarrat MA, Ibrahim G, Taha F, Al Tamimi A, Shukur M, Elmoselhi AB (2019). The correlation between smart device usage & sleep quality among UAE residents. Sleep Med.

[18] Jaber N, Oudah M, Kowatli A (2016). Dietary and lifestyle factors associated with dyspepsia among pre-clinical medical students in Ajman, United Arab Emirates. Cent Asian J Glob Health.

[19] Jacob S, Tambawel J, Trooshi FM, Alkhoury Y ( 2013). Consumption pattern of nutritional health drinks and energy drinks among university students in Ajman, UAE. Gulf Medical Journal.

[20] Al-Houqani M, Leinberger-Jabari A, Al Naeemi A (2018). Patterns of tobacco use in the United Arab Emirates Healthy Future (UAEHFS) pilot study. PLoS One.

[21] Mutare S, Feehan J, Cheikh Ismail L (2022). The first United Arab Emirates national representative birth cohort study: study protocol. Front Pediatr.

[22] Loney T, Aw TC, Handysides DG (2013). An analysis of the health status of the United Arab Emirates: the 'Big 4' public health issues. Glob Health Action.

[23] Mezhal F, Oulhaj A, Abdulle A (2021). The interrelationship and accumulation of cardiometabolic risk factors amongst young adults in the United Arab Emirates: the UAE Healthy Future Study. Diabetol Metab Syndr.

[24] Shah SM, Loney T, Sheek-Hussein M (2015). Hypertension prevalence, awareness, treatment, and control, in male South Asian immigrants in the United Arab Emirates: a cross-sectional study. BMC Cardiovasc Disord.

[25] World Health Organization (2018). Global action plan on physical activity 2018-2030: more active people for a healthier world. Geneva.

[26] Alrahma AM, Al Suwaidi H, AlGurg R (2023). Results from the United Arab Emirates 2022 report card on physical activity for children and adolescents. J Exerc Sci Fit.

[27] Paulo MS, Nauman J, Abdulle A (2018). Results from the United Arab Emirates’ 2018 report card on physical activity for children and youth. J Phys Act Health.

[28] Al-Rifai RH, Majeed M, Qambar MA, Ibrahim A, AlYammahi KM, Aziz F (2019). Type 2 diabetes and pre-diabetes mellitus: a systematic review and meta-analysis of prevalence studies in women of childbearing age in the Middle East and North Africa, 2000-2018. Syst Rev.

[29] Kalan Farmanfarma KH, Ansari-Moghaddam A, Zareban I, Adineh HA (2020). Prevalence of type 2 diabetes in Middle-East: Systematic review & meta-analysis. Prim Care Diabetes.

[30] Saadi H, Al-Kaabi J, Benbarka M (2010). Prevalence of undiagnosed diabetes and quality of care in diabetic patients followed at primary and tertiary clinics in Abu Dhabi, United Arab Emirates. Rev Diabet Stud.

